# Health Promotion at Local Level in Norway: The Use of Public Health Coordinators and Health Overviews to Promote Fair Distribution Among Social Groups

**DOI:** 10.15171/ijhpm.2018.22

**Published:** 2018-03-14

**Authors:** Susanne Hagen, Kjell Ivar Øvergård, Marit Helgesen, Elisabeth Fosse, Steffen Torp

**Affiliations:** ^1^Department of Health, Social and Welfare Studies, University College of Southeast Norway, Kongsberg, Norway.; ^2^Department of Maritime Operations, University College of Southeast Norway, Kongsberg, Norway.; ^3^Institute for Urban and Regional Research, OsloMet – Oslo Metropolitan University, Oslo, Norway.; ^4^Department of Health Promotion and Development, University of Bergen, Bergen, Norway.

**Keywords:** Equity, HiAP, Public Health Coordinator, Norway, Health Promotion

## Abstract

**Background:** Norway is internationally known today for its political and socio-economic prioritization of equity. The 2012 Public Health Act (PHA) aimed to further equity in the domain of health by addressing the social gradient in health. The PHA’s main policy measures were (1) delegation to the municipal level of responsibility for identifying and targeting underserved groups and (2) the imposition on municipalities of a "Health in All Policies" (HiAP) approach where local policy-making generally is considered in light of public health impact. In addition, the act recommended municipalities employ a public health coordinator (PHC) and required a development of an overview of their citizens’ health to reveal underserved social segments. This study investigates the relationship between changes in municipal use of HiAP tools (PHC and health overviews) with regard to the PHA implementation and municipal prioritization of fair distribution of social and economic resources among social groups.

**Methods:** Data from two surveys, conducted in 2011 and 2014, were merged with official register data. All Norwegian municipalities were included (N=428). Descriptive statistics as well as bi- and multivariate logistic regression analyses were performed.

**Results:** Thirty-eight percent of the municipalities reported they generally considered fair distribution among social groups in local policy-making, while 70% considered fair distribution in their local health promotion initiatives. Developing health overviews after the PHA’s implementation was positively associated with prioritizing fair distribution in political decision-making (odds ratio [OR] = 2.54; CI: 1.12-5.76), compared to municipalities that had not developed such overviews. However, the employment of PHCs after the implementation was negatively associated with prioritizing fair distribution in local health promotion initiatives (OR = 0.22; CI: 0.05-0.90), compared to municipalities without that position.

**Conclusion:** Development of health overviews — as requested by the PHA — may contribute to prioritization of fair distribution among social groups with regard to the social determinants of health at the local level.

## Background

Equity in health is an overall goal — globally, nationally, and locally.^1^ Yet health inequalities, significantly related to socio-economic status, persist within and between countries, and by some measures and in some places, are increasing.^2^ Those inequalities can be mapped on a gradient — shaped by the distribution of the social determinants of health, related in turn to distribution of money, power, and resources as determined by political choices at all levels.^1,3-5^ For decades, social equity in health has been an explicit political goal in the Scandinavian welfare states.^6^ Norway, epitomizes that model, with its emphasis on solidarity, broad redistributive policies, and fair distribution of services, but nevertheless has recently evidenced increased inequalities in health.^7-9^ This in turn has provoked new policy responses, particularly since 2000, to raise the health status of groups less well-off so as to level the health gradient.^10^

“Health in All Policies” (HiAP) is one such response, stipulating that health impacts be taken into account in all areas of policy-making. HiAP encourages coordination and collaboration (both *horizontally* across various sectors and *vertically* among authorities at local, regional, and national levels) to achieve high levels of public health broadly shared.^11,12^ HiAP aims to foster an explicit health focus through the “whole of government,” with policies to support and improve public health,^13^ which thus is seen as a broad governmental responsibility rather than for the health sector alone.^14^ Norway, among other countries, has implemented the HiAP approach in national and local public health policy.^15^

In 2012, the Norwegian Public Health Act (PHA) took effect, based on policies of health equity, HiAP, sustainable development, the precautionary principle, knowledge-based approaches, and civil-society participation.^16^ The act aimed to be a state-of-art summation of welfare state ideals in the domain of public health. In particular, it aimed to serve responsiveness and systematicity by shifting decision-making to the local level.^17^ The PHA states that fair distribution of resources is the basis of good public health policy, and makes levelling up the social gradient in health a key focus by acting on the social determinants of health.^18^ Socio-economic factors — such as income and wealth, education, employment and working circumstances, housing and food security, and health and social services — are together responsible for many health inequalities.^1,19^ With the HiAP approach, involving multiple stakeholders at all political levels,^11^ the PHA gives clear roles and objectives for the state, county, and municipal government levels. But it is at the municipal level that the PHA gives overall responsibility for the population’s health and public health work.^16,18^ So the actions and policies of Norwegian municipalities play an important and demanding role in reducing health inequalities,^20^ and fair distribution of resources at local level are essential with regard to reducing the health gradient.^21^

Norway’s 428 municipalities, while differing sometimes radically in size and geography, provide to their citizens the main bulk of welfare services: primary health care, schooling, care for children and the elderly, social support and services, culture, agriculture, and socio-economic development.^22^ In addition to implementing national welfare policies and addressing public health, municipalities constitute democratic entities making decisions based on local realities, needs, and preferences. HiAP argues for the impact on health being systematically evaluated in developing and implementing local policies in all areas.^11,12^ To accomplish this, the PHA requires municipalities to develop a local health overview of positive and negative factors determining health in their populations.^16^ The health overview is supposed to identify local health challenges, with a special focus on health inequalities. This information should then become the basis for prioritizing municipal planning and decision-making.^23^ The ideal is to anchor public health in municipal policy-making. In particular, the PHA recommends that municipalities employ a public health coordinator (PHC) to facilitate collaboration and coordination across all sectors.^18^ For securing involvement in local policy-making, initiatives and municipal planning, it is recommended that the PHC is employed in a position close to full-time and situated high in the political chain of command, for example within the office of the chief executive officer.^24-26^ Despite this, it has been documented that the PHCs often are employed in low part-time positions, and not close to the municipal policy-making, for example within the office of medical officer in the municipal.^22^

As a model of social-democratic policy, Norway’s experience at implementing HiAP, via the 2012 PHA, and using municipal PHCs and health overviews has potential relevance beyond its own borders, for any policy seeking more equitable public health outcomes. The aim of this study was to explore awareness of equity at the local level by investigating the relationship between municipal use of PHCs and health overviews (defined as HiAP tools) *and* fair distribution of the social determinants of health in the municipalities. Our research questions were:

1) Has municipal use of PHCs and the development of health overviews changed after the enactment of the PHA?
2) To what extent do municipalities prioritize fair distribution among social groups in political decision-making *and* in their local health promotion initiatives?
3) Are changes in municipal use of PHCs and health overviews associated with municipal prioritization of fair distribution among social groups in political decision-making *and* in local health promotion initiatives?

## Methods

This study is part of the SODEMIFA project exploring how health promotion policy addresses social inequalities in health in Norwegian.

### Research Design

The study has a cross-sectional design with elements of natural experiment, the latter made possible by measuring municipalities’ use of PHC and health overviews before and after implementation of the PHA. Our data derive from surveys exploring local public health practice and official registries from Statistic Norway (SSB) and the Norwegian Social Sciences data services (NSD).

### Data Collection

We used data from two questionnaires, one from a baseline study conducted in 2011 and one from a post PHA-implementation survey conducted in 2014. In general, the two questionnaires used in, respectively, 2011 and 2014 concerned the same topics, but some questions were formulated differently. Both questionnaires were evaluated for content validity by using relevant literature, reviews by relevant professionals, and comparison with similar surveys. Datasets from the questionnaires were merged with the register data by using the unique identification code of the municipalities.

### Sample

All Norwegian municipalities (N = 428) were included in both surveys. Both questionnaires were sent electronically to the official address of the municipalities, addressed to the chief executive officer. A total of 58% of the municipalities completed the entire baseline questionnaire, whereas 87% responded to parts of it. The post PHA-implementation questionnaire was completed by 61% of the municipalities, while 75% answered to parts of it. The data from the registries were more or less complete (n = 427).

### Variables

#### Municipal Change in Use of Public Health Coordinator

To reflect change of municipal use of HiAP tools, we included questions concerning the use of PHCs and the development of health overviews from both the baseline survey (2011) and the post-intervention survey (2014). The question regarding PHCs was asked the same way in both questionnaires: “Does the municipality have a PHC?” The response alternatives were: “yes” (1), “no” (2), and “do not know” (3). We recoded these variables into “no/do not know” (0) and “yes” (1).

The data on use of PHCs in 2011 and 2014 were merged to construct the following categories: “had both before and after enactment of law” (1), “acquired after enactment of law” (2), “removed after enactment of law” (3), and “never had” (4). Since we were only concerned with municipalities’ use of HiAP tools, we recoded these into three categories: “had both before and after enactment of law” (1), “acquired after enactment of law” (2), and “removed after enactment/never had” (3).

#### Municipal Change in Development of Health Overviews

The question concerning development of health overviews was also formulated the same way both times: “Has the municipality developed an overview of inhabitants’ health status, and the positive and negative determinants of health?” The response alternatives differed in the questionnaires. In 2011, the response categories were: “yes” (1), “no” (2), and “do not know” (3). We recoded these alternatives into “no/do not know” (0), and “yes” (1). In 2014, response alternatives were: “yes” (1), “no” (2), “we are about to start up this work” (3), and “do not know” (4). For this variable, we recoded the alternatives into: “no/we are about to start up this work/do not know” (0), and “yes” (1).

Based upon this recoding, we merged the 2011 and 2014 data to identify the following categories: “had both before and after enactment of law” (1), “acquired after enactment of law” (2), “removed after enactment of law” (3), and “never had” (4). Since we were only concerned with municipalities’ use of HiAP tools, we recoded these into three categories: “had both before and after enactment of law” (1), “acquired after enactment of law” (2), and “removed after enactment/never had” (3).

#### Variables of Local Health in All Policies Factors

We included four variables from the 2014 questionnaire reflecting local use of HiAP approaches. The *first variable* was whether the municipality had “strengthened the competence base for health promotion” (answers “no” [= 0], or “yes” [= 1]). The *second variable* was whether the municipality had “increased collaboration with voluntary organizations” (possible answers where “no” [= 0], or “yes” [= 1]). The *third variable* was: “Does the municipality collaborate with external actors in health promotion networks?” Response alternatives were, “yes”, “no”, and “do not know” – which were recoded into “no/do not know” = (0), and “yes” = (1). The *fourth variable* was “Has the municipality established cross-sectorial working groups for health promotion at the strategic level?” Possible answers were “yes,” “no,” “we are about to start up this work,” and “do not know” – which were recoded into “no/we are about to start up this work/do not know” = (0), and “yes” = (1).

#### Municipal Background Variables

Register data from 2014 regarding municipal size and centrality were included. The size of the municipalities (SSB) was categorized into five groups: <3000 inhabitants (0), 3000-4999 inhabitants (1), 5000-9999 inhabitants (2), 10 000-34 999 inhabitants (3) and ≥35 000 inhabitants (4).

The measurement of municipal centrality is based on Standard Classification of Municipalities (SSB) and defines a municipality’s geographical location in relation to a larger city with higher central functions. Norway is a sparsely populated country, and centrality is defined by the time it takes to travel to the nearest centre. We define a municipality as a centre if, among other criteria, it has public institutions, a wide range of public and private services, and to which it is possible to commute. If it takes more than 90 minutes to drive to the nearest centre, a municipality is located peripherally. Centrality was categorized into four categories from less central (=0) to most central (=3).

#### Municipal Prioritization of Fair Distribution (Dependent Variable)

Fair distribution among social groups with regard to economic and social resources is essential to level the health gradient, and the post-implementation survey from 2014 was concerned with the municipality’s stated prioritization of fair distribution. We included two questions considering this theme, were the first variable was concerned with fair distribution in the municipal policy-making processes, while the latter variable was concerned with fair distribution in local health promotion initiatives. The first included question was: “Are considerations of fair distribution a priority in political decision-making?” The response categories were: “yes” (1), “no” (2), and “do not know” (3). We recoded the variables into “no/do not know” (0), and “yes” (1). The second question was, “Are considerations of fair distribution a priority in the area of local health promotion initiatives?” The response categories were the same as for the previous question, and we recoded this variable similarly.

### Statistical Analyses

Descriptive analyses were performed for all the included variables (Tables 1 and 2). When checking for high inter-correlation (>0.7)^27^ by use of Pearson’s *r*, we did not reveal any multicollinearity between the variables.

**Table 1:** Descriptive Data of Municipal Change in Use of PHC and Health Overview With Regard to the Implementation of the Norwegian PHA

	**No. (%)**
PHC^a^ (n = 210)	
Had both before and after enactment	146 (70)
Acquired after enactment	33 (16)
Removed after enactment / never had	31 (14)
Development of health overview^b^ (n = 168)	
Had both before and after enactment	20 (12)
Acquired after enactment	50 (30)
Removed after enactment/never had	98 (58)

Abbreviations: PHA, Public Health Act; PHC, public health coordinator. ^a^ Based on municipal employment of PHCs *in 2011* (n = 332) – yes: 252 (76%); no: 80, (24%); and *in 2014* (n = 275) – yes: 234 (85%); no: 41 (15%). ^b^ Based on development of health overviews Norwegian municipalities *in 2011* (n = 296) – yes: 53 (18%); no: 243 (82%); and *in 2014* (n = 276) – yes: 105 (38%); no: 171 (62%).

**Table 2:** Descriptive Data on Local HiAP Factors, Background Variables, and Municipal Prioritization of Fair Distribution Among Social Groups in Norwegian Municipalities

**Factors**	**No. ( %) **	**Mean ± SD**
*Local HiAP factors*		
Strengthen competence base of health promotion (n = 428)		
No	223 (52)	
Yes	205 (48)	
Increased collaboration with voluntary organizations (n = 428)		
No	281 (66)	
Yes	147 (34)	
Collaboration with external actors (n = 272)		
No	76 (28)	
Yes	196 (72)	
Cross-sectorial working groups at strategic level (n = 273)		
No	105 (39)	
Yes	168 (61)	
*Background variables of the municipalities*		
Size (n = 427)		2.40 ± 1.32
<3000 inhabitants	158 (37)	
3000-4999 inhabitants	70 (16)	
5000-9999 inhabitants	86 (20)	
10 000-34 999 inhabitants	90 (21)	
≥35 000 inhabitants	23 (5)	
Centrality (n = 427)		1.53±1.29
0	149 (35)	
1	51 (12)	
2	77 (18)	
3	150 (35)	
*Municipal prioritizing of fair distribution among social groups*		
Fair distribution in political decision-making (n = 254)		
No	158 (62)	
Yes	96 (38)	
Fair distribution in local health promotion initiatives (n = 257)		
No	77 (30)	
Yes	180 (70)	

Abbreviation: HiAP, Health in All Policies.

We checked whether the distribution of municipalities’ centrality and size in the sample (N = 155) reflected the distribution in the population (N = 427). Table 3 shows the distribution in the population and the sample for these two variables as well as the calculated weights for each variable.

**Table 3:** Calculated Weights for Centrality and Size

**Centrality**	**Size**						
**Code**	**Population Frequency (Proportion)**	**Sample Frequency (Proportion)**	***W***_cent_	**Code**	**Population Frequency (Proportion)**	**Sample Frequency (Proportion)**	***W***_size_
1	149 (0.349)	56 (0.364)	0.959	1	158 (0.370)	44 (0.286)	1.295
2	51 (0.119)	17 (0.110)	1.082	2	70 (0.164)	19 (0.123)	1.329
3	77 (0.180)	31 (0.201)	0.896	3	86 (0.201)	34 (0.221)	0.912
4	150 (0.351)	50 (0.325)	1.080	4	90 (0.211)	43 (0.279)	0.755
				5	23 (0.054)	14 (0.091)	0.593

Notes: Weights for centrality (*w*_cent_) and size (*w*_size_) are calculated by dividing the population proportion by the sample proportion. The combined weight for size and centrality is calculated by multiplying the two weights (*w*_cent_ * *w*_size_) for each municipality.

Bi- and multi-variate logistic regression analyses were used to investigate associations between the variables of change in PHC, change in health overview, variables of local HiAP factors, municipal background variables, *and* the variables reflecting prioritization of fair distribution.

All multivariate analyses were done in a hierarchical manner. First, we included the variables of change in PHC and health overview for both dependent variables (Table 4, Model A & Table 5, Model A) to investigate the association between these tools and the local prioritization of fair distribution among social groups. Then we controlled for the local HiAP factors and background variables (Table 4, Model B & Table 5, Model B). The strength of associations is presented as odds ratio (OR) with 95% CI.

**Table 4:** Logistic Regression Analyses for Fair Distribution Among Social Groups in Political Decision-Making

**Factors**	**Fair Distribution Among Social Groups in Political Decision-Making**		
	**Bivariate** **OR (95% CI)**	**Multivariate, Model A** **OR (95% CI), n = 155**	**Multivariate, Model B** **OR (95 % CI), n = 155**
**Municipal changes in use of HiAP tools**			
***PHC***			
Removed after enactment/never had	1.00	1.00	1.00
Had both before and after enactment	1.23 (0.51-2.97)	0.82 (0.29-2.31)	0.42 (0.13-1.38)
Acquired after enactment	1.26 (0.43-3.65)	0.68 (0.19-2.37)	0.41 (0.10-1.64)
***Development of health overview***			
Removed after enactment/never had	1.00	1.00	1.00
Had both before and after enactment	3.74 (1.27-11.02)^a^	3.85 (1.29-11.46)^a^	2.42 (0.72-8.06)
Acquired after enactment	2.49 (1.20-5.18)^a^	2.60 (1.24-5.45)^a^	2.54 (1.12-5.76)^a^
**Local HiAP factors**			
Strengthen competence base for health promotion	1.58 (0.86-2.87)		1.30 (0.54-3.13)
Increased collaboration with voluntary organizations	1.44 (0.87-2.40)		1.89 (0.86-4.14)
Collaboration with external actors	2.30 (1.28-4.36)^a^		2.70 (1.08-6.79)^a^
Cross-sectorial working groups at strategic level	1.91 (1.21-3.26)^a^		1.89 (0.75-3.41)
**Municipal background variables**			
Size	1.24 (1.02-1.52)^a^		1.78 (1.21-2.62)^a^
Centrality	0.98 (0.80-1.19)		0.66 (0.44-0.95)^a^

Abbreviations: HiAP, Health in All Policies Factors; PHC, public health coordinator; OR, odds ratio. Notes: All analyses were weighted with a combing weight of size and centrality. ^a^ Significant associations.

**Table 5:** Logistic Regression for Fair Distribution Among Social Groups in Local Health Promotion Initiatives

**Factors**	**Fair Distribution Among Social Groups in Local Health Promotion Initiatives**		
	**Bivariate** **OR (95% CI)**	**Multivariate, Model A** **OR (95% CI), n = 155**	**Multivariate, Model B** **OR (95 % CI), n = 155**
**Municipal changes in use of HiAP tools**			
*PHC*			
Removed after enactment/never had	1.00	1.00	1.00
Had both before and after enactment	1.41 (0.59-3.34)	1.14 (0.39-3.28)	0.75 (0.22-2.58)
Acquired after enactment	0.51 (0.18-1.43)	0.41 (0.12-1.41)	0.22 (0.05-0.90)
*Development of health overview*			
Removed after enactment/never had	1.00	1.00	1.00
Had both before and after enactment	2.42 (0.64-9.15)	2.31 (0.60-8.91)	1.37 (0.31-6.04)
Acquired after enactment	2.39 (1.10-5.21)^a^	2.65 (1.18-5.93)^a^	2.18 (0.90-5.28)
**Local HiAP factors**			
Strengthen competence base for health promotion	2.51 (1.42-4.45)^a^		2.95 (1.30-6.72)^a^
Increased collaboration with voluntary organizations	1.81 (1.07-3.04)^a^		1.11 (0.50-2.49)
Collaboration with external actors	1.80 (1.04-3.14)^a^		2.98 (1.28-6.94)^a^
Cross-sectorial working groups at strategic level	1.43 (0.85-2.42)		1.38 (0.63-2.98)
**Municipal background variables**			
Size	1.25 (1.01-1.54)^a^		1.51 (1.03-2.22)^a^
Centrality	1.10 (0.90-1.35)		0.85 (0.57-1.24)

Abbreviations: HiAP, Health in All Policies Factors; PHC, public health coordinator; OR, odds ratio. Notes: All analyses were weighted with a combing weight of size and centrality. ^a^ Significant associations.

Sample size in the multi-variate analyses differed because of the unequal response rates to the questions in the datasets from 2011 and 2014. Regarding this, we had no options for substituting missing variables in Model A for both regressions, but in Model B we substituted missing independent variables, except from the variables that measure change.

All statistical analyses were performed with the IBM Statistics Program for Social Sciences SPSS v22.0 computer package (IBM Corp., Armonk, NY, USA) for the statistical analyses. The significance level was set at *P* < .05 and all tests were two sided.

## Results

### Descriptions

#### Change of Public Health Coordinator

A total of 70% of the municipalities had employed a PHC position both before and after the enactment of the PHA, while 16% established this position after the act took effect. Only a minority of municipalities (14%) had never employed a PHC-position, or had removed it after the PHA-enactment (Table 1).

#### Change in Health Overview

Only 12% of the municipalities had developed a health overview before the PHA-enactment, whereas 30% had made such an overview after. However, a large proportion of Norwegian municipalities (58%) had not developed an overview of their citizens’ health as a tool to be used in their local health promotion work (Table 1).

#### Local Health in All Policies factors

Nearly half (48%) of the Norwegian municipalities reported that they had strengthened their health promotion competence. With regard to collaboration, about one third (32%) of the municipalities had increased the collaboration with voluntary organizations, whereupon 72% of the municipalities had collaborated with external actors and 61% had made use of cross sectional working groups at strategic level (Table 2).

#### Prioritization of Fair Distribution

Less than half of the municipalities (38%) reported that they prioritized fair distribution among social groups in local political decision-making, while 70% of the municipalities prioritized fair distribution among social groups in their local health promotion initiatives (Table 2).

### Associations Between PHC and Health Overview and Prioritization of Fair Distribution in Political Decision-Making

The bivariate analyses (Table 4) show that municipalities developing overviews both before and after the PHA had much higher prioritization of fair distribution among social groups in political decision-making (OR = 3.74; CI: 1.27-11.02) than those municipalities that never had health overviews. A similar positive association was also found for those municipalities that developed a health overview after the enactment of the PHA (OR = 2.49; CI: 1.20-5.18) compared to municipalities that never had health overviews. Three other co-variates also had statistical significant and positive bivariate effects: municipalities that had “collaboration with external actors” (OR = 2.30; CI: 1.28-4.36), municipalities that had “cross-sectional working groups” (OR = 1.91; CI: 1.21-3.26), and “size of the municipalities” (OR = 1.24; CI: 1.02-1.52).

For multivariate logistic analyses (Table 4, Model A), we found that developing health overviews had statistically significant positive associations with prioritizing fair distribution among social groups in political decision-making in the municipality, compared to municipalities not having developed health overviews (health overviews developed before and after OR = 3.85; CI: 1.29-11.46, developed after the enactment of PHA, OR = 2.60; CI: 1.24-5.45).

When including additional covariates (Table 4, Model B), only the statistically significant association regarding municipalities that had acquired a health overview after the PHA-enactment (OR = 2.54; CI: 1.12-5.76) was retained. Collaboration with external actors (OR= 2.70; CI: 1.08-6.79), municipal size (OR = 1.78; CI: 1.21-2.62) and the centrality of the municipalities (OR = 0.66; CI: 0.44-0.95) were also significantly associated with municipalities prioritizing fair distribution among social groups in municipal political decision-making.

### Associations Between PHC and Health Overview and Local Health Promotion Initiatives

Bivariate logistic analyses showed that several variables were significantly associated with municipalities prioritizing fair distribution among social groups in local health promotion initiatives (Table 5): Municipalities that acquired health overviews after the PHA took effect (OR = 2.39; CI: 1.10-5.21), municipalities that had strengthened their competence base on health promotion (OR = 2.51; CI: 1.42-4.45), increased their collaboration with voluntary organizations (OR = 1.81; CI: 1.07-3.04), municipalities that had collaboration with external actors (OR = 1.80; CI: 1.04-3.14), and municipalities being larger-sized (OR = 1.25; CI: 1.01-1.54) each showed all statistically significant associations with local health promotion initiatives.

Results from the multivariate logistic analyses (see Table 5, Model A) show that municipalities having acquired the health overview after the act was enforced (OR = 2.65; CI: 1.18-5.93) were significantly associated with prioritizing fair distribution among social groups in health promotion initiatives, compared to municipalities not having developed such overview.

When including all the variables (Table 5, Model B), this significant association was not retained. However, acquiring a PHC position after the enactment was significantly negatively associated (OR = 0.22; CI: 0.05-0.90) with prioritizing fair distribution among social groups in health promotion initiatives, compared with municipalities not having such position. Strengthening of the competence base (OR = 2.95; CI: 1.30-6.72), collaboration with external actors (OR = 2.98; CI: 1.28-6.94), and increasing municipal size (OR = 1.51; CI: 1.03-2.22), were all significantly positively associated with municipalities prioritizing fair distribution among social groups in local health promotion initiatives.

## Discussion

Tackling health inequalities is a goal for Norwegian health promotion policy, and is seen to be linked to fair distribution among social groups of socio-economic resources at the local level.^18^ Norway’s 2012 PHA aimed to reduce health inequalities, and this study sheds light on equity at the local level and municipal use of HiAP tools encouraged by the PHA. Our study found that the PHCs are widely employed in the municipalities, and most of them before the enactment of the PHA. Relatively few municipalities have developed health overviews, but compared to the number of municipalities having such overviews before the PHA, the increase is rather high.

Only 38% of the municipalities prioritized fair distribution among social groups in political decision-making, and municipalities having developed health overviews after the PHA’s enactment were two and a half times more likely to prioritize fair distribution compared to municipalities who had not developed such overviews. Seventy percent of the municipalities reported that they took into account fair distribution in planning local health promotion initiatives. Municipalities focused on strengthening their health promotion competence and establishing networks for collaboration with external actors were almost three times more likely to take fair distribution into account, compared to municipalities that did not. Our study has also found that municipalities having employed PHCs after the act was enforced were almost four times *less likely* to prioritize fair distribution in local health promotion initiatives compared to the municipalities not having employed such a coordinator.

### Implementation of the Norwegian Public Health Act and Municipal Change in Use of HiAP Tools

Our study identified changes in municipal use of HiAP tools possibly due to the implementation of the PHA (Table 1). Our results indicate only a small increase of 16% in municipal employment of PHCs — a finding that does not surprise, since our results show that almost three-quarters of Norwegian municipalities had employed a PHC before the enactment and continued to make use of this position after the enforcement. This finding is in line with a study showing that some years before the implementation, 62% of municipalities in Norway had employed such a function for coordination.^24^ During the early 2000s, Norway revitalized health promotion policies nationally, and the policy encouraged municipalities to establish positions for PHCs. Research has suggested that especially provision of funds from central governments to municipalities for a PHC,^25^ but also enhanced municipal health promotion projects, increase likelihood of the PHC position being established.^28^ With our study showing the considerable majority of municipalities making use of a PHC (86%), one may conclude that Norwegian municipalities have largely accommodated the recommendation made by national policies.

Health overviews — encouraged by national policy in Norway since 1987^17^ and required by the PHA — aim to map out the health situation of a municipality’s citizens, and corresponding positive and negative determinants influencing health, as a basis for local planning and policy decisions.^15,23^ Our study shows that only 12% of the municipalities undertook health overviews before the enactment of the PHA, a proportion that increased to 42% after the act took effect. The relative large increase in adoption after the PHA arguably shows its influence on municipalities. Nevertheless, our study shows that more than half of Norwegian municipalities (58%) have not crafted such a document, probably meaning they are “flying blind” as they make policy and allocate resources without a map to citizens’ health status and corresponding determinants. Research has indicated that developing health overviews is both ambitious and demanding.^15^ Among other factors, the complexity of the determinants, access to information, and lack of knowledge may complicate this work.

Although our study documents that though relevant HiAP tools are being used in local health promotion in Norway, there is still a question of how much these tools are being brought to bear on fair distribution of socio-economic resources among social groups at the local level.

### Municipal Focus on Fair Distribution Among Social Groups in Norway

#### Prioritizing of Fair Distribution Among Social Groups in Political Decision-Making at Local Level

Our study found that about one-third of Norwegian municipalities reported that they prioritized fair distribution among social groups in municipal political decision-making (Table 2). Based on national guidelines — particularly those of the PHA — that number seems surprisingly low.^12,13,18^ Are a majority of municipalities in Norway simply throwing up their hands, overwhelmed by the task? Certainly, there has been a debate to what degree levelling the health gradient by action on social determinants at the local level is actually possible. Health determinants, as first described by Dahlgren and Whitehead,^29,30^ arise at different politico-administrative levels — but mostly levels beyond those of individual municipalities. The broad determinants — national and regional labor markets, national fiscal and tax policies, as well as educational and health policies — are mainly decided nationally and internationally.^2,31^ In countries such as the Scandinavian welfare states, in which municipalities have had for a long time already major public health functions and responsibilities,^32^ municipalities serve as agents for the welfare state by implementing national policies and are policy-makers in their own right, ideally in dynamic response to changing local realities. Herein lies their potentially important contribution to levelling the health gradient. However, other studies have noted that reducing inequalities may be challenging for municipalities because of cross-sectorial requirements and lack of municipal competencies.^20,22^ In addition, challenges with regard to collaboration and coordination^33^ among relevant actors may be another constraint.

This study identified that municipalities developing health overviews *after* the act took effect were two and a half times more likely to prioritize fair distribution in political decision-making compared to municipalities that had never developed such overviews (Table 4). This result may indicate that the very process of developing a municipal health overview fosters the institutional awareness, engagement, and the will to level the gradient that the overview helps bring to light. If so, this result suggests, further, that the PHA has succeeded to some degree in bringing national policy to the local level, which an earlier study, before the act was enforced, found had happened only to a small degree.^22^ In principle, municipalities do not themselves decide what services to provide, but they do decide how to organize and provision them. In addressing health inequalities, this requires municipalities to choose among competing policy approaches. For instance, Hanssen and Helgesen^34^ claim that, to have a fundamental effect on levelling the health gradient, high-quality services should be provided universally rather than to targeted groups only. By contrast, Marmot^3^ and Carey and Grammond,^35^ in pursuit of the same goal, argue for a “proportionate universalism” in which universal services are combined with efforts aimed at disadvantaged groups. To the extent municipalities opt for the latter approach, they need to positively select individuals and groups to whom extra services are to be provided. The competencies to detect disadvantage must be present, as must the resources to address the need. Given that it is often challenging and costly for municipalities to develop services in the needed direction, an overview of factors determining citizens’ health is both a necessary tool and, in the very crafting, part of the process of strengthening local competencies. Yet these undertakings are often new to planning and to policy making at the municipal level.^15^ Integrating the public health perspective into public policy — not to mention fair distribution — is challenging.

Nevertheless, we found that municipalities collaborating with external actors were almost three times more likely to prioritize fair distribution in political decision-making. This result is perhaps not surprising as levelling the gradient is collaborative in its nature and requires “whole of government” approach. Other studies have suggested that eg, county councils and county governors have significant influence on municipalities in their strive for levelling the gradient in health.^25,36^ We also found that larger municipalities — and, contrastingly, less central ones — were more likely to prioritize fair distribution in municipal decision-making.

#### Prioritizing of Fair Distribution Among Social Groups in Local Health Promotion Initiative

A very large proportion of Norwegian municipalities prioritized consideration of fair distribution among social groups in local health promotion initiatives (Table 2). At first sight, this is a result in line with the Norwegian national public health policy,^18,37^ but precisely how prioritization is manifested in the overall municipal policy-making process invites closer analysis. Kiland and colleagues^38^ identified two contrasting approaches existing side by side in the Norwegian public health policy arena. One approach reflects a collective-integration perspective, focusing on the social determinants of health and seeking equity in health through overall municipal planning and cross-sectorial collaboration. By contrast, a rival approach focuses on empowering individuals to take responsibility for their own health.^38^ The latter approach is easier to adopt at the local level because it is simpler to work with individual health behavior issues, in contrast to structural factors.^39^ One manifestation of municipalities’ preference for individual health behavior issues over collective/structural approaches is municipal “healthy-lifestyle” centers, which have become more and more popular in Norway, with an increase from 42 in 2008, to 212 in 2014.^38^ Yet with the Norwegian welfare state’s long focus on disadvantaged groups,^40^ the individual health behavior approach has not completely dominated in public health policies. Many municipalities make special efforts, in the mode of “proportionate universalism,” to care for the less well off without necessarily developing knowledge bases for prioritization and policy-making.

Several studies suggest that Norwegian local health promotion initiatives most often arise from bureaucratic enthusiasm, emerging from a single sector or unit rather than from an overall assessment of identified challenges, and suffer as a result from being only weakly anchored in the municipal apparatus as a whole.^22,25,39^ A national audit showed that 86% of municipalities have initiated public health initiatives, with only 35% of the initiatives being universal, 19% of them directed to vulnerable groups, and 41% a mix of both.^41^ While our study show that 70% of the municipalities emphasize fair distribution in local health promotion initiatives, perhaps the majority of these initiatives focus on specific individual behaviors in targeted groups, and lack a systemic and structural focus. One risk of piecemeal initiatives directed towards the most vulnerable is that they may help narrow the health gap in specific areas, but do not necessarily contribute much to levelling the overall health gradient.^3,35^ Several studies have identified that it is not easy to understand the issues of health equity and the gradient adequately, and the public health field is struggling with gaining this insight.^21,42^

Our results show that prioritizing fair distribution among social groups in local health promotion initiatives is associated with a strengthened competence base of public health and with collaboration with external actors (Table 5). Our result leads us to suggest that municipalities focusing on equity in health are strengthening their competence to act, but that policy responses so far tend to cash out as single, targeted initiatives, with only narrow bureaucratic support. This is suboptimal in light of evidence indicating that a more effective approach shifts local public-health efforts in a collective-integration direction supported with a wider cross-sectorial footprint. Not only more effective programs can result from a broader approach, but also increased understanding of the social determinants of health. For instance, a recent study found almost 82% of Norwegian municipalities queried believed they were capable of reduce inequalities in health, but only half acknowledged the importance of living conditions, which has been highlighted as an essential factor to address when reducing inequalities.^43^

Our study found that municipalities employing PHCs after the act was implemented were almost four times *less likely* to prioritize fair distribution in local health promotion initiatives compared to municipalities not having a PHC. At first glance, this is a surprising result because this coordinative function has long been recommended in Norway as one of the most important HiAP tool,^18,44^ and our result might indicate that the PHCs counteract such prioritization. Earlier studies have indicated that the PHCs play an essential role in levelling the gradient,^39,45^ but perhaps municipalities having employed PHCs after the act took effect consider themselves as satisfied with just having accommodated the recommendation in the PHA, and have lagged in efforts to refine that function for achieving its potential and mandate. Moreover, the PHA gave local governments — and not the health sector alone — responsibility for public health and reducing inequalities in health. Perhaps municipalities not having employed PHCs have an integrative-collaborative culture and are not in need for such a coordinative function. Local public health should be organized and administrated from the staff of the chief executive officer^46^ and the PHA clearly gives the chief executive officer this responsibility.^16^ From this point of view, perhaps the intention of the PHA regarding inter sectorial collaboration for health may have been fulfilled in these municipalities without a PHC. These municipalities seem to perform better with regard to equality than municipalities employing PHCs after the act took effect.

Nevertheless, the result discussed above raises another reflection: have PHCs not been the success we had reason to believe? Critical voices have questioned the design of the PHC position in municipalities, arguing that the share of positions is too small and the tasks accorded to the role often unclear. Further, some contend that the position is organizationally remote from where municipal decisions are actually made, with PHCs having at best uncertain influence on actual health promotion coordination.^47,48^ In addition, the skills for which PHCs are selected are not clear, nor whether they are the competencies needed to motivate for social equity in health. Our results suggest a need for greater understanding of the PHC’s role in effectively coordinating public health work to ensure the broad goal of HiAP. With respect to PHC effectiveness, one study found that PHCs needed to be in a part-time position of at least 70% of full-time employment to adequately fulfill the tasks of coordinating, planning, and liaising,^26^ while another study suggested those roles required a 90% of full-time position.^48^ Average PHC position in Norway in 2014 was 34% of full-time.^38^ If cross-sectorial working groups and collaboration with external actors emanates bottom-up from a felt need, there may be possibilities for a PHC, even short of full-time, to work successfully, catalyzing relationships and effectively linking public and non-governmental actors. But where municipal health policies tend to be formulated and imposed in a top-down manner, a PHC in a limited part-time position, and localized in a service-providing unit, will be weakly positioned to coordinate policies and inter-sectorial activities, even while potentially able to spearhead single health promotion measures.

## Limitations

This study has a cross-sectional design, and therefore any conclusions about causality cannot be drawn. Moreover, for both included surveys in this study, the smallest and the less central municipalities were those that responded to a lesser degree.^45,47^ In addition, we had a low response rate for the change variables calculated from both the 2011 and 2014 data sets. The sample distribution derivatived from the population distribution for municipal size and partly for the centrality, but we estimated weights for these derivations and used these estimations in weighted binary logistic regressions analyses. The low N in our study lead to rather large variance of the estimated parameters and thereby resulting in wide confidence intervals, large standard errors and low statistical power. The consequence of this is a probability for not identifying statistical associations that may exist in the dataset (Type II-error).^49^ Nevertheless, this is the first time data from two sets have been merged in the Norwegian context in order to examine the associations between the use of HiAP tools and equity awareness at the local level. We believe our results are valuable for the practice and research field. Future research should strive to attend a higher N than was obtained in our study. To strengthen the knowledge base in this field additionally, one may combine solid quantitative methods with qualitative in-depth approaches using a mixed method design.

Another limitation of our study may be that the surveys were sent electronically to an official e-mail account, addressed to the chief executive officer. The topic of health promotion practices is complex and demands competent respondents with insight and understanding, especially on the questions related to fair distribution. By sending the questionnaire to the chief executive officer, who has the overall responsibility for health promotion in the municipalities,^16^ we assume the survey has been answered by the most knowledgeable respondents, but we cannot be sure how often this was not the case. For the 2014 survey, we know that the respondents were mainly chief executive officers, PHCs, and heads of units for health care, all persons expected to have insight in the field of health promotion. For the 2011 survey, we do not know who answered the questionnaires, but we expect the respondents to be more or less the same persons or persons holding the same positions as those responding in the 2014 survey. Moreover, based on previous comparable surveys,^24^ the response seem trustworthy. Overall, a limitation that applies to responding to surveys in general is the tendency to respond positively or provide the information the respondents interpret as the aim of the survey,^50^ but according to the discussion above, we must rely on that our responses are trustworthy.

## Conclusion

In line with the recommendations and requirements of Norway’s 2012 PHA, this study shows that Norwegian municipalities are increasingly applying HiAP tools, such as creating a PHC position and crafting municipal health overviews. With municipal PHCs long urged by the national government, the PHA’s enactment brought about only a small increase in their number, but the PHA sparked a significant increase in municipalities developing health overviews. We found fair distribution of the social determinants of health among social groups to be a significant focus of municipal health promotion initiatives, but to a smaller degree a focus of municipal policy-making in general. Analysis of our data showed relationships between municipalities having developed health overview after the implementation of PHA and prioritizing fair distribution among social groups in municipal decision-making: municipalities that developed health overviews after the act took effect were two and a half times more likely to prioritize fair distribution in political decision-making compared to municipalities that did not develop such overviews.

Previous research had identified a gap between national goals in Norway and local public health policy, but our findings suggest that this gap is narrowing after the implementation of the PHA. Our findings support evidence of progress toward the nationally prescribed goal of levelling the health gradient in Norway.

Further research can identify the connection between different forms of health governance at state and municipal levels, including the HiAP approach, and fair distribution at the local level. A fruitful way of exploring this complex field could be mixed methods to investigate the unanswered questions both in extension and in-depth.

## Acknowledgements

This study was founded by The Norwegian Research Council [grant numbers: 213841/H10, 229628/H10]. Thanks to Professor Jens B. Grøgaard, School of Business, Department of Business, History and Social Sciences, University College of Southeast Norway, for statistical advices.

## Ethical issues

The Norwegian Social Science Data Services (NSD, Bergen, Norway) approved this study.

## Competing interests

Authors declare that they have no competing interests.

## Authors’ contributions

The leading author is SH. KIØ and ST has contributed in the data analyses, while all authors have contributed in the manuscript draft. All authors approved the manuscript. EF and MH were responsible for the SODEMIFA project and data collecting.

## Authors’ affiliations

^1^Department of Health, Social and Welfare Studies, University College of Southeast Norway, Kongsberg, Norway. ^2^Department of Maritime Operations, University College of Southeast Norway, Kongsberg, Norway. ^3^Institute for Urban and Regional Research, OsloMet – Oslo Metropolitan University, Oslo, Norway. ^4^Department of Health Promotion and Development, University of Bergen, Bergen, Norway.

## Key messages

**Implications for policy makers**

- The very process of developing a health overview seems to build the institutional muscle, awareness, and skills among relevant municipal personnel to address health inequalities.
- Employing a public health coordinator (PHC) does not necessarily lead to greater focus on equity.
- For PHCs to succeed in reducing health inequalities they need necessary information and competencies, to be employed in positions close to full-time, and possess sufficient organizational authority to coordinate municipal sectors and assist in developing and implementing policies.

**Implications for the public**

Attaining more equitable public health requires coordination of national and local policy. Our research suggests that tasking municipalities with developing local health overviews of the social determinants of health can help build both municipal awareness and competencies for addressing inequalities. A tentative unexpected finding is that merely employing a public health coordinator (PHC) can reduce the capacity to advance equity. If the PHC position is only part- time, and not empowered enough or bureaucratically integrated, or held by someone skilled at building needed cross-sectorial collaboration, equity might not be advanced.
